# Need for Routine Brain Magnetic Resonance Imaging for Unilateral Facial Palsy in Emergency Department

**DOI:** 10.3390/diagnostics15172135

**Published:** 2025-08-24

**Authors:** Hanna Park, Youn-Jung Kim, Won Young Kim

**Affiliations:** Department of Emergency Medicine, Asan Medical Center, University of Ulsan College of Medicine, Seoul 05505, Republic of Korea; phn0915@amc.seoul.kr (H.P.); yjkim.em@amc.seoul.kr (Y.-J.K.)

**Keywords:** Bell’s palsy, facial paralysis, magnetic resonance imaging, emergency service, hospital

## Abstract

**Objectives:** The need for routine brain magnetic resonance imaging (MRI) for patients presenting with unilateral facial palsy in the emergency department (ED) is a subject of ongoing debate. This study aimed to evaluate the diagnostic yield of MRI in this population and to identify clinical risk factors associated with non-idiopathic causes, to inform selective imaging strategies. **Methods:** This single-center, retrospective study was conducted in the ED of a tertiary-care center in Korea. We analyzed adult patients (aged ≥ 18 years) who presented with facial palsy as the primary symptom between 1 January 2020 and 31 December 2022. Patients with other neurological abnormalities detected during the initial examination or those who did not undergo brain MRI were excluded. The primary outcome was the identification of positive MRI findings, defined as brain lesions (e.g., ischemic stroke, tumor, and hemorrhage) considered causally related to the facial palsy based on anatomical correlation and radiological interpretation. Patients were categorized into positive or negative MRI groups accordingly, and baseline characteristics were compared between the groups. **Results:** Among the 436 patients who underwent brain MRI, 13 (3.0%) showed positive findings such as brain tumors or stroke that led to diagnoses other than Bell’s palsy, while the remaining 423 (97.0%) were ultimately diagnosed with Bell’s palsy. The proportion of patients with a history of transient ischemic attack/stroke and malignancy was significantly higher in the group with non-idiopathic facial palsy (*p* = 0.02 and *p* < 0.001, respectively). **Conclusions:** In adults presenting to the ED with clinically isolated unilateral facial palsy and no other neurological signs, routine brain MRI had a low diagnostic yield (3%). A history of malignancy or prior TIA/stroke was associated with alternative diagnoses. A selective imaging strategy based on risk factors may improve diagnostic efficiency without compromising safety.

## 1. Introduction

Bell’s palsy is the most common peripheral palsy of the seventh cranial nerve, with affected patients typically presenting with rapid onset of unilateral facial weakness or paralysis [1,2,3]. Bell’s palsy is typically a diagnosis of exclusion and often managed on the basis of the clinical presentation when no other neurological deficits are present. In cases where patients exhibit typical features of Bell’s palsy, additional laboratory or radiological examinations are generally not required [4,5,6]. Although it is often a benign disease with symptoms and signs resolving within 6 months, facial palsy can be associated with less common conditions such as stroke, neoplasm, infection, or pontine hemorrhage [7,8,9,10,11].

The need to perform brain magnetic resonance imaging (MRI) for patients presenting with facial palsy remains a subject of ongoing debate. While routine MRI may be a standard procedure to rule out secondary causes in some settings, it may not be necessary for patients without additional neurological symptoms. From an economical perspective, the necessity of MRI for the diagnosis of Bell’s palsy should be carefully considered [12].

Current clinical guidelines vary [4,5,6], and it remains unclear whether routine MRI adds value to the diagnostic process or unnecessarily increases healthcare costs and resource utilization.

To address this gap, we investigated patients who presented with clinically isolated unilateral facial palsy and underwent brain MRI in the emergency department (ED) in order to evaluate the diagnostic yield of brain MRI. By analyzing the MRI results of these patients, we aimed to establish the necessity of routine MRI for confirming the diagnosis of Bell’s palsy and determine if it should be reserved for cases with specific clinical indications in ED.

## 2. Methods

### 2.1. Study Design and Participants

This single-center, retrospective study included adult patients (aged ≥18 years) who presented to the ED with facial palsy as the primary symptom from 1 January 2020 to 31 December 2022. Patients were excluded if they exhibited neurological abnormalities other than facial palsy during the initial examination or did not undergo MRI. The study protocol was approved by the institutional review board of our hospital (study no. 2023-0916), and the requirement for informed consent was waived due to the retrospective design. All procedures were conducted in accordance with the Declaration of Helsinki and local data privacy regulations. This study is an observational study conducted using an existing database and was carried out in accordance with the STROBE statement.

### 2.2. Variables

Data were extracted from medical records of the included patients. Demographic information, including sex, age, primary symptoms, and time of symptom onset, was collected. Comorbid conditions such as hypertension, diabetes, and a history of transient ischemic attack (TIA) or stroke were also recorded. Neurological findings were based on the initial assessment performed by emergency physicians (residents or attending physicians), and included the House−Brackmann facial nerve grading scale and other relevant neurological signs. All patients who presented to the ED with neurologic complaints underwent a structured neurological examination, which was recorded in the electronic medical record.

In accordance with the institutional protocol, physicians are required to assess and document at least four of the following seven neurological domains to qualify for procedural billing under the “simple neurologic examination” code: (1) level of consciousness and higher cortical function, (2) cranial nerve function, (3) limb motor strength, (4) limb sensory function, (5) deep tendon reflexes, (6) cerebellar and vestibular function, and (7) gait and balance.

For the purpose of this study, “clinically isolated unilateral facial palsy” was defined as unilateral facial weakness without any other documented neurological abnormalities on initial examination. MRI results were reviewed on the basis of the interpretations provided by a radiology specialist. All brain MRIs during the study period were performed using the same 1.5-Tesla scanner (MAGNETOM Avanto, Siemens Healthineers, Erlangen, Germany).

### 2.3. Outcomes

The primary outcome was the presence of positive MRI findings. Patients were categorized into two groups based on MRI results: those with positive findings and those with negative findings. An MRI was considered positive if it revealed a brain lesion deemed causally related to the facial palsy (e.g., ischemic stroke, tumor, and hemorrhage), based on anatomical correlation and interpretation by a board-certified radiologist. Findings unrelated to facial nerve pathways or considered incidental were classified as negative. In cases where brain MRI revealed structural abnormalities, the relevance of these findings to the patient’s presenting facial palsy was determined through consultation with a board-certified neurologist or neurosurgeon. If the lesion was deemed anatomically unrelated, contralateral to the symptoms or not clinically compatible with acute onset facial palsy, it was classified as incidental. These patients were ultimately diagnosed with idiopathic (Bell’s) palsy based on the specialist’s judgment. Radiologic enhancement of the facial nerve alone, in the absence of an alternative diagnosis, was not regarded as a positive finding. Baseline characteristics were compared between the two groups.

### 2.4. Statistical Analysis

Continuous variables are presented as mean with standard deviations or median with interquartile range (IQR), depending on distribution. Categorical variables are reported as number and percentage (%). Continuous variables were compared using the unpaired Student’s *t*-test for normally distributed data and the Mann–Whitney U test for non-normally distributed data. Categorical variables were compared using the chi-square test. To explore the factors associated with non-idiopathic facial palsy, univariate logistic regression was first performed. Variables with statistical significance were entered into a multivariable logistic regression model. Given the limited number of outcome events (*n* = 13), the events-per-variable (EPV) for our multivariable model was approximately 4.3. We acknowledge that this falls below the recommended threshold of 10 and have the risk of overfitting. We performed sensitivity analysis of Firth’s penalized likelihood logistic regression to account for small sample size and rare events. Odds ratios (ORs) were determined using logistic regression analysis with a 95% confidence interval (CI). All statistical analyses were performed using IBM SPSS Statistics, version 21 (IBM Corp., Armonk, NY, USA) and R software, version 4.5.1 (R Foundation for Statistical Computing, Vienna, Austria).

## 3. Results

During the study period, 630 adult patients presented to the ED with facial palsy as the chief complaint. We excluded 51 patients without objective paralysis on neurological examination, 37 with additional neurological deficits, and 29 with pre-existing brain lesions or prior Botox injections. This left 513 patients with clinically isolated unilateral facial palsy, of whom 77 did not undergo brain MRI based on clinical judgment and were excluded from further analysis.

**Figure 1:**
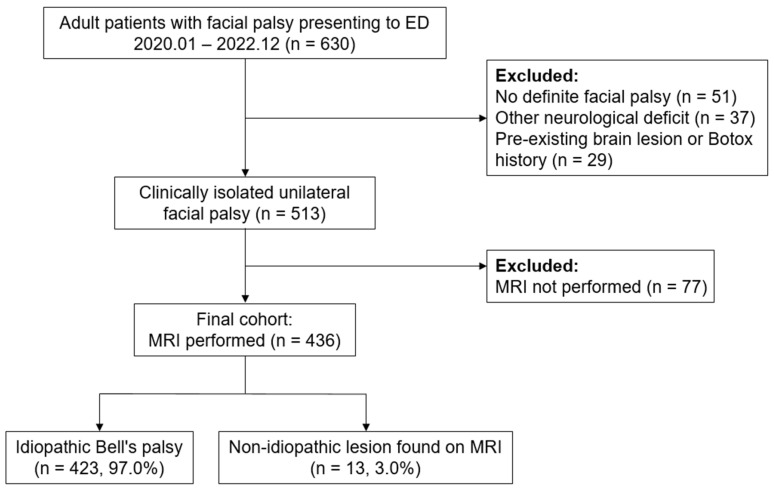
Patient flow diagram. ED—emergency department; MRI—magnetic resonance imaging.

The final cohort included 436 patients who underwent brain MRI (Figure 1). Among them, 13 (3.0%) had non-idiopathic lesions considered causative of their facial palsy (seven brain tumors or lymphomas, five ischemic strokes, and one pontine hemorrhage). Of the remaining 423 patients, eight had incidental findings (e.g., brain tumors, subdural hemorrhage, or infarction) deemed unrelated to their facial palsy after specialist review. These patients were ultimately diagnosed with Bell’s palsy.

Table 1 summarizes the baseline characteristics of the enrolled patients. The mean age of patients was 58 years, and 52.3% were women. The mean time from symptom onset to the ED presentation was 25 h. In total, 41.3% patients exhibited facial palsy with House–Brackmann grades 4–5. No significant differences were found between the Bell’s palsy group and the non-idiopathic facial palsy group in terms of these demographic and clinical characteristics. A history of TIA/stroke (*p* = 0.02) and active malignancy (*p* < 0.001) were significantly more frequent in the non-idiopathic facial palsy group.

Diffusion-weighted MRI was performed for 92% patients; 8% underwent MRI with contrast enhancement. In the Bell’s palsy group, 98.1% patients received a consultation with ear, nose, and throat (ENT) specialists, and the median ED stay was approximately 283 min. However, in the other facial palsy group, the median duration of the ED stay was 626 min.

Table 2 presents the results of logistic regression analyses. Univariate logistic regression analysis showed that the time of onset and the presence of comorbid diseases such as TIA/stroke and malignancy were significant factors, with ORs of 4.23 (95% CI: 1.10 to 16.26), 0.04 (95% CI: 1.10 to 16.26), and 18.57 (95% CI: 5.81 to 59.42), respectively. In multivariate logistic regression analysis that included variables with statistical significance in univariate analysis, the presence of TIA/stroke and active malignancy remained significant, with adjusted ORs of 12.08 (95% CI: 2.26 to 64.41) and 27.08 (6.32 to 116.08), respectively. To account for the small number of events and potential overfitting, we additionally performed Firth’s penalized logistic regression as a sensitivity analysis. The results were consistent in direction and magnitude, supporting the robustness of our findings. Full results are presented in Supplementary Table S1.

**Figure 2:**
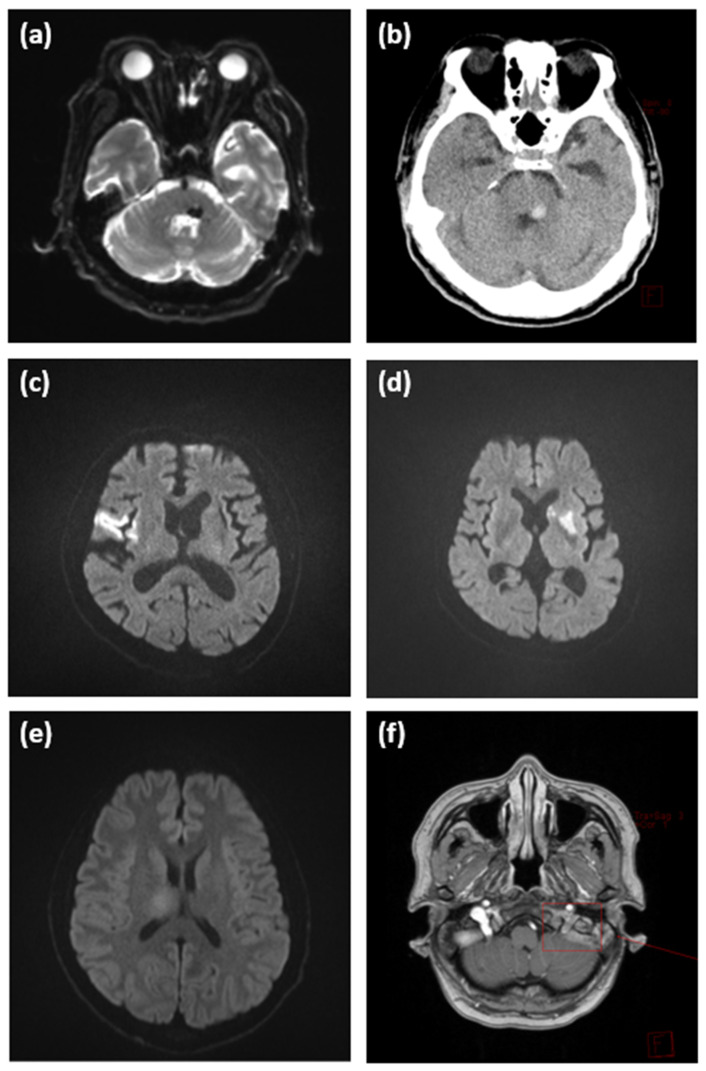
Brain lesions detected in patients presenting with facial palsy. (**a**) Diffusion-weighted MRI showing acute hemorrhage in the left pons (Case #1). (**b**) Non-contrast axial CT scan of the brain, obtained after diffusion-weighted MRI (**a**), with a high-density window setting (WL = 1054, WW = 95), showing a hyperdense pontine lesion consistent with hemorrhage (Case #1). (**c**) Diffusion-weighted MRI showing acute infarction in the right middle cerebral artery territory (Case #2). (**d**) Diffusion-weighted MRI showing acute infarction in the left basal ganglia and corona radiata (Case #7). (**e**) Diffusion-weighted MRI showing a mass-like lesion in the right thalamus, suspected to be a brain tumor (Case #12). (**f**) Contrast-enhanced 3D T1-weighted axial MRI (gadolinium phase) showing brain metastasis involving the left jugular foramen (Case #9). The red box and arrow indicate the enhancing lesion at the left jugular foramen. The lesion was not clearly visible on diffusion-weighted imaging. MRI—magnetic resonance imaging; CT—computed tomography; MCA—middle cerebral artery.

Table 3 and Figure 2 detail the positive MRI findings observed in the 13 patients whose final diagnosis differed from idiopathic Bell’s palsy. Brain tumors included newly diagnosed primary brain neoplasms, skull or brain parenchymal metastases from systemic malignancy, and leptomeningeal seeding. Ischemic strokes were found in the middle cerebral artery (MCA) territory, basal ganglia, internal capsule, corona radiata, and posterior circulation. One case involved pontine hemorrhage with an underlying cavernous malformation.

In each case, the anatomical relevance of the lesion to the patient’s presenting facial palsy was evaluated by board-certified neurologists, neurosurgeons, or oncologists. While most lesions clearly corresponded to known facial nerve pathways or brainstem structures, in two cases (Cases #7 and #12), the correlation was less direct. Nevertheless, after multidisciplinary consultation, these lesions were considered clinically relevant to the facial palsy and thus classified as positive MRI findings.

## 4. Discussions

During the study period, 3% patients with unilateral facial palsy underwent brain MRI and exhibited positive findings that correlated with their symptoms, including brain tumors, ischemic stroke, and pontine hemorrhage. This indicates the presence of cases where initial neurological examination alone may not be sufficient for accurate diagnosis.

Facial palsy may result from either central or peripheral lesions. Central lesions, such as strokes involving the motor cortex or corticobulbar tract, typically spare the forehead. In contrast, peripheral causes—including Bell’s palsy, tumors, infections, or trauma—affect the facial nerve nucleus or its distal course and often present with complete unilateral facial weakness. Recognizing these patterns is important for accurate diagnosis.

The diagnosis of Bell’s palsy is generally based on clinical examination alone, without the need for laboratory tests or imaging studies. However, the 2020 guideline from the French Society of ENT suggests, based on expert opinion, that MRI may be warranted in cases with atypical presentations in order to differentiate between Bell’s palsy and other conditions like stroke [5]. Given the existence of several reports of cases, such as those of occult tumors or pontine strokes mimicking Bell’s palsy and leading to misdiagnosis [13,14,15,16,17,18,19], there is a need to understand the frequency with which different diagnoses are established for patients who primarily exhibit facial palsy.

In this study, among 513 patients with clinically isolated unilateral facial palsy, 436 underwent MRI. This reflected both patient preferences and the high imaging rates intended to avoid misdiagnoses in tertiary hospitals. Among the 436 patients, 21 showed positive MRI findings. Of these, eight were considered incidental and unrelated to Bell’s palsy. The remaining 13 patients required reevaluation of the initial diagnosis. For example, for patient #1, initial examination was limited because of dementia; however, MRI revealed an infarct in the MCA territory. Patient #13 initially complained of facial palsy only during the initial examination, although MRI detected pontine hemorrhage. Further imaging with brain computed tomography confirmed the presence of a lesion correlating with the symptoms. Subsequent examinations revealed additional symptoms such as diplopia, nystagmus, extraocular muscle limitation, and dysarthria, which had not been reported initially. These cases revealed the challenges faced while diagnosing Bell’s palsy solely on the basis of the patient’s reported symptoms, especially when initial neurological examinations are limited or unreliable.

Additionally, patients #7 to #11 had active malignancies and underwent MRI for exclusion of brain metastasis, despite the absence of other abnormalities in the neurological examination. This further underscores the utility of MRI in cases where malignancy is a concern.

Thus, while MRI may be useful for diagnosing conditions that mimic Bell’s palsy, it may not be necessary for all patients with clinically isolated unilateral facial palsy if initial neurological evaluations are thorough and do not indicate other risk factors or neurological signs. A selective imaging strategy based on risk factors such as malignancy or TIA/stroke may improve diagnostic efficiency without compromising safety.

In support of this selective imaging strategy, we estimated the diagnostic yield and number needed to image (NNI) for different clinical subgroups. In the overall cohort of 436 patients, the NNI to identify one clinically relevant brain lesion was approximately 33.5. However, among patients with either a history of malignancy or prior TIA/stroke (*n* = 63), 10 had clinically relevant findings, resulting in an NNI of just 6.3. These results suggest that risk factor-based triage can substantially improve diagnostic efficiency and reduce resource use in the ED. The corresponding estimates are summarized in Supplementary Table S2.

**Figure 3:**
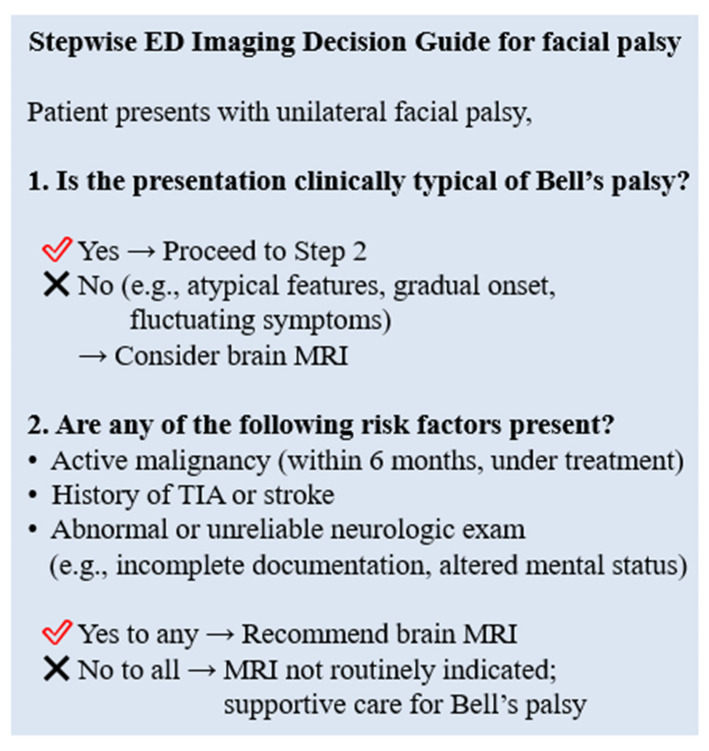
Stepwise emergency department imaging algorithm for unilateral facial palsy. MRI—magnetic resonance imaging; TIA—transient ischemic attack.

To translate these findings into clinical practice, we developed a stepwise ED imaging decision guide (Figure 3). This guide incorporates both clinical presentation and patient-specific risk factors, such as active malignancy, prior TIA/stroke, or unreliable neurological examination. While not intended as an absolute protocol, it may serve as a practical tool to assist clinicians in making more efficient and targeted decisions regarding brain MRI in patients presenting with facial palsy.

## 5. Limitations

This study has several limitations. First, its retrospective design relied on chart documentation, which may have been incomplete or inconsistent, potentially leading to misclassification of clinically isolated facial palsy. Second, as a single-center study in a tertiary ED with high MRI availability and a patient population enriched with comorbidities, both selection and work-up bias may have influenced both MRI utilization and lesion detection rates. Imaging protocols were not standardized, and higher-risk or atypical patients were more likely to undergo contrast-enhanced imaging. The timing between symptom onset and MRI acquisition was also variable, which may have affected lesion detectability, particularly for ischemic or enhancing lesions. Third, only 13 patients had non-idiopathic facial palsy, resulting in a low EPV (4.3) and potential overfitting in multivariable analysis, despite sensitivity analyses using penalized regression. Finally, the high accessibility of MRI and the relatively high prevalence of malignancy in our cohort may limit the generalizability of these findings to community or resource-limited settings. These factors may have increased the positive predictive value of selective imaging strategies in this population. Therefore, our results should be interpreted with caution when applied to populations in other countries or healthcare environments.

## 6. Conclusions

In adults presenting to the ED with clinically isolated unilateral facial palsy and no additional neurological signs, the diagnostic yield of routine brain MRI was low (3%). A history of active malignancy or prior TIA/stroke appeared to increase the likelihood of alternative diagnoses. These findings suggest that a selective imaging approach—based on individual risk factors and the reliability of the initial neurological examination—may help optimize resource utilization while maintaining diagnostic safety.

## Figures and Tables

**Table 1:** Baseline characteristics of patients with clinically isolated unilateral facial palsy according to MRI results (idiopathic Bell’s palsy vs. non-idiopathic lesions).

Characteristics	Final Cohort	Idiopathic Bell’s Palsy	Non-Idiopathic Lesion on MRI	*p*-Value
	*N* = 436	*N* = 423 (97.0%)	*N* = 13 (3.0%)	
Age (years) (median, IQR)	58 (47–66)	58 (47–66)	56 (48.5–76.5)	0.48
Sex (female)	228 (52.3%)	224 (53.0%)	4 (30.8%)	0.16
Past medical history				
Hypertension	136 (31.2%)	130 (30.7%)	6 (46.2%)	0.24
Diabetes	96 (22.0%)	94 (22.2%)	2 (15.4%)	0.56
Dyslipidemia	72 (16.5%)	71 (16.8%)	1 (7.7%)	0.38
TIA/stroke	31 (7.1%)	28 (6.6%)	3 (23.1%)	0.02
Neurological disease other than CVA	8 (1.8%)	7 (1.7%)	1 (7.7%)	0.11
Malignancy	32 (7.3%)	25 (5.9%)	7 (53.8%)	<0.001
Current pregnancy status	1 (0.2%)	1 (0.2%)	0 (0)	0.86
Previous Bell’s palsy	23 (5.3%)	23 (5.4%)	0 (0)	0.39
Onset (hours before) (median, IQR)	25 (10–48)	24 (10–48)	24 (9–204)	0.36
HB grade				
Grades 2–3	241 (55.3%)	234 (55.4%)	7 (43.9%)	
Grades 4–5	180 (41.3%)	177 (41.8%)	3 (23.1%)	0.41
ENT consultation	416 (95.4%)	415 (98.1%)	1 (7.7%)	<0.001
MR modality				
DWI	401 (92.0%)	396 (93.6%)	5 (38.5%)	
With enhance	35 (8.0%)	27 (6.4%)	8 (61.5%)	<0.001
Time spent in ED (min) (median, IQR)	289 (217.25–392.75)	283 (215.00–381.00)	626 (473.00–853.00)	<0.001

IQR—interquartile range; TIA—transient ischemic attack; CVA—cerebrovascular accident; HB—House−Brackmann; ENT—ear, nose, and throat department; MR—magnetic resonance; DWI—diffusion-weighted image; ED—emergency department.

**Table 2:** Logistic regression analysis of risk factors for non-idiopathic lesions in patients with clinically isolated unilateral facial palsy.

Variables	Univariate	Multivariate
	OR (95% CI)	AOR (95% CI)
Age (years) (median, IQR)	1.02 (0.98–1.06)	
Sex (female)	0.40 (0.12–1.30)	
Hypertension	1.93 (0.64–5.86)	
Diabetes	0.64 (0.14–2.92)	
Dyslipidemia	0.41 (0.05–3.23)	
TIA/stroke	4.23 (1.10–16.26)	12.08 (2.26–64.41)
Neurological disease other than CVA	4.95 (0.56–43.48)	
Malignancy	18.57 (5.81–59.42)	27.08 (6.32–116.08)
Onset (hours before) (median, IQR)	1.01 (1.00–1.01)	1.00 (1.00–1.01)
HB grades 4–5	0.57 (0.14–2.22)	

IQR—interquartile range; TIA—transient ischemic attack; CVA—cerebrovascular accident; HB— House−Brackmann; OR—odds ratio; AOR—adjusted odds ratio; CI—confidence interva

**Table 3:** Characteristics of patients with non-idiopathic facial palsy: positive MRI findings.

Case	Age	Sex	Symptoms	Comorbid Disease	Final Diagnosis	MRI Findings
1	76	M	Lt. facial palsy	HTN, DM, stroke	brain hemorrhage	Recent hemorrhage, underlying cavernous malformation in the left pons.
2	82	M	Lt. facial palsy	HTN, DM, stroke, Dementia	Ischemic stroke	Acute infarction in right MCA territory.
3	79	M	Lt. facial palsy	HTN, DL	Ischemic stroke	Acute infarction, right internal capsule posterior limb and corona radiata.
4	69	M	Lt. facial palsy	HTN, Malignancy	Ischemic stroke	Multifocal acute infarction at right frontal and right insular lobes, the distal territory of superior division of right MCA.
5	56	F	Rt. Facial palsy	Malignancy	Brain tumor	Extensive heterogeneously enhancing lesion involving the clivus, both sphenoid bones, right petrous bone, and right occipital condyle with involvement of right Meckel’s cave.
6	63	M	Rt. Facial palsy	HTN, stroke	Ischemic stroke	Multifocal acute embolic infarctions in the posterior circulation territory.
7	77	F	Rt. Facial palsy	HTN	Ischemic stroke	Acute infarction in the left basal ganglia and corona radiata.
8	47	F	Rt. Facial palsy	Malignancy	Brain tumor	Diffuse pachymeningeal and bone metastases.
9	50	F	Rt. Facial palsy	Malignancy	Brain tumor	Several bone metastases in the central to posterior skull base, left parietal bone, covered upper cervical spine, and, suspiciously, right parietal bone. Involvement of the left jugular foramen, possibly causing lower cranial nerve palsies with obliteration of the left internal jugular vein.
10	46	M	Rt. Facial palsy	Malignancy	Brain tumor	Leptomeningeal pathology such as meningitis or CSF seeding.
11	52	M	Lt. facial palsy	Malignancy	Brain tumor	Newly noted multiple metastases with/without perilesional edema in the bilateral cerebral hemispheres.
12	20	M	Lt. facial palsy	None	Brain tumor	Marginally increased size of T2 hyperintense lesion in the right thalamus (r/o glioma).
13	54	M	Rt. Facial palsy	Malignancy	Brain tumor	Newly appeared multifocal enhancing lesions in the calvaria, skull base, scanned cervical vertebrae, right frontal scalp, and dura meter along the right inferior temporal lobe, with suspicious invasion of right CN VII, VIII, and right petrosal sinus.

HTN—hypertension; DM—diabetes mellitus; MCA—middle cerebral artery; DL—dyslipidemia; CSF—cerebrospinal fluid; CN—cranial nerve

## Data Availability

The data presented in this study are available on request from the corresponding author. The data are not publicly available due to privacy and ethical restrictions.

## Supplementary Materials

The following supporting information can be downloaded at: https://www.mdpi.com/article/10.3390/diagnostics15172135/s1, Table S1: Results of Firth’s penalized logistic regression for predictors of positive MRI findings; Table S2: Diagnostic yield and number needed to image (NNI) for clinically relevant brain lesions in patients with clinically isolated facial palsy.

## References

[1] Basic-Kes V., Dobrota V.D., Cesarik M., Matovina L.Z., Madzar Z., Zavoreo I., Demarin V. (2013). Peripheral facial weakness (Bell’s palsy). Acta Clin. Croat..

[2] Holland N.J., Bernstein J.M. (2014). Bell’s palsy. BMJ Clin. Evid..

[3] Warner M.J., Hutchison J., Varacallo M. (2023). Bell Palsy. StatPearls.

[4] Baugh R.F., Basura G.J., Ishii L.E., Schwartz S.R., Drumheller C.M., Burkholder R., Deckard N.A., Dawson C., Driscoll C., Gillespie M.B. (2013). Clinical Practice Guideline: Bell’s Palsy. Otolaryngol.–Head Neck Surg..

[5] Fieux M., Franco-Vidal V., Devic P., Bricaire F., Charpiot A., Darrouzet V., Denoix L., Gatignol P., Guevara N., Montava M. (2020). French Society of ENT (SFORL) guidelines. Management of acute Bell’s palsy. Eur. Ann. Otorhinolaryngol. Head Neck Dis..

[6] de Almeida J.R., Guyatt G.H., Sud S., Dorion J., Hill M.D., Kolber M.R., Lea J., Reg S.L., Somogyi B.K., Westerberg B.D. (2014). Management of Bell palsy: Clinical practice guideline. CMAJ.

[7] Heckmann J.G., Urban P.P., Pitz S., Guntinas-Lichius O., Gagyor I. (2019). The Diagnosis and Treatment of Idiopathic Facial Paresis (Bell’s Palsy). Dtsch. Arztebl. Int..

[8] Zimmermann J., Jesse S., Kassubek J., Pinkhardt E., Ludolph A.C. (2019). Differential diagnosis of peripheral facial nerve palsy: A retrospective clinical, MRI and CSF-based study. J. Neurol..

[9] May M., Klein S.R. (1991). Differential diagnosis of facial nerve palsy. Otolaryngol. Clin. N. Am..

[10] George E., Richie M.B., Glastonbury C.M. (2020). Facial Nerve Palsy: Clinical Practice and Cognitive Errors. Am. J. Med..

[11] Fahimi J., Navi B.B., Kamel H. (2014). Potential misdiagnoses of Bell’s palsy in the emergency department. Ann. Emerg. Med..

[12] Kazemian E., Schaffer H.M., Wozniak A., Leonetti J.P. (2022). Economic Impact of Diagnostic Imaging in the Workup of Uncomplicated Bell’s Palsy. J. Neurol. Surg. B Skull Base.

[13] Dunphy L., Kaur R., Flossmann E. (2021). Pontine stroke mimicking Bell’s palsy: A cautionary tale!. BMJ Case Rep..

[14] Karadan U., Manappallil R.G., Jayakrishnan C., Supreeth R.N. (2018). Pontine haemorrhage disguised as Bell’s palsy. BMJ Case Rep..

[15] Mabel H.M., Othman N.B., Cheah W.K. (2022). Pontine stroke: A rare mimicker of Bell’s palsy. Med. J. Malays..

[16] Mower S. (2017). Bell’s palsy: Excluding serious illness in urgent and emergency care settings. Emerg. Nurse.

[17] Yao L., Wang B., Lu F., He X., Lu G., Zhang S. (2023). Facial nerve in skullbase tumors: Imaging and clinical relevance. Eur. J. Med. Res..

[18] Chung E.J., Matic D., Fung K., MacNeil S.D., Nichols A.C., Kiwan R., Tay K., Yoo J. (2022). Bell’s palsy misdiagnosis: Characteristics of occult tumors causing facial paralysis. J. Otolaryngol. Head Neck Surg..

[19] Nguyen C.N., Mallepally N., Tabilona J.R., Lu L.B. (2021). Not So Benign Bell’s Palsy: Malignant Peripheral Nerve Sheath Tumor of the Facial Nerve Involving the Temporal Bone. J. Gen. Intern. Med..

