# Supplementary material for: Need for Routine Brain Magnetic Resonance Imaging for Unilateral Facial Palsy in Emergency Department
Source: Diagnostics (Basel). 2025 Aug 24;15(17):2135. doi: 10.3390/diagnostics15172135 (PMC12428370; doi:10.3390/diagnostics15172135)
Supplement: Supplementary file 1 [file diagnostics-15-02135-s001.zip › diagnostics-3758184-supplementary.pdf]

Table S1. Results of Firth's Penalized Logistic Regression for Predictors of Positive MRI Findings

Variable	Coefficient (β)	Standard Error	Odds Ratio (OR)	95% CI (Lower – Upper)	p-value
Intercept	–4.662	0.537	–	–	<0.001
TIA/Stroke	2.565	0.781	13.0	2.65 – 63.9	0.0028
Malignancy	3.438	0.679	31.2	8.69 – 134.6	<0.001

Model details:

Firth's penalized likelihood logistic regression was performed using two key variables (TIA/Stroke and Malignancy), identified as significant predictors of positive brain MRI findings in univariate analysis. The model accounts for the low number of events (n = 13) and potential small-sample bias. The direction and magnitude of the associations remained consistent with the conventional multivariable model, supporting the robustness of the study findings.

Table S2. Diagnostic Yield and Number Needed to Image (NNI) for Clinically Relevant Brain Lesions in Patients with Clinically Isolated Facial Palsy

Group	Number of MRIs performed	Number of clinically relevant lesions detected	Detection rate (%)	Number Needed to Image (NNI)
All patients with clinically isolated facial palsy (no risk stratification)	436	13	3	33.5
Patients with risk factors (malignancy or TIA/stroke)	63	10	15.9	6.3

This table summarizes the diagnostic yield of brain MRI in detecting non-idiopathic lesions among patients presenting with clinically isolated unilateral facial palsy.

We observed that among all 436 patients who underwent MRI, 13 (3.0%) were found to have clinically relevant brain lesions. This corresponds to a Number Needed to Image (NNI) of 33.5—meaning that 33.5 MRI scans were required to detect one significant lesion.

However, when limiting the analysis to the subgroup of patients with at least one predefined clinical risk factor (either a history of malignancy or prior TIA/stroke), the diagnostic yield increased markedly. In this high-risk group (n = 63), 10 patients (15.9%) had relevant lesions, corresponding to an NNI of 6.3.

These findings suggest that a selective imaging strategy targeting high-risk individuals could improve diagnostic efficiency and may offer better cost-effectiveness in emergency department settings.
